# Does a cannibal feeding strategy impart differential metabolic performance in young burbot (*Lota lota maculosa*)?

**DOI:** 10.1093/conphys/coaa034

**Published:** 2020-05-03

**Authors:** Amanda J Frazier, Nathan R Jensen, Shawn P Young, Anne E Todgham

**Affiliations:** 1 Department of Animal Science, University of California Davis, Davis, CA 95616, USA; 2 Kootenai Tribe of Idaho, Bonners Ferry, ID 83805, USA

**Keywords:** Aquaculture, burbot, cannibalism, conservation hatchery, feeding strategy, metabolic performance

## Abstract

The practice of mitigating cannibalism in aquaculture is an important focus for hatcheries seeking to maximize yield and has been maintained in hatcheries focusing on wild stock restoration. We hypothesize, however, that a cannibal feeding strategy may confer performance advantages over a non-cannibal feeding strategy and that perhaps cannibal size grading may not be optimal for hatcheries focusing on conservation goals. This study examined metabolic performance differences between cannibal and non-cannibal burbot, *Lota lota maculosa*, at the Kootenai Tribe of Idaho Twin Rivers Hatchery in Moyie Springs, ID, USA. After habitat alteration led to functional extinction of burbot in the region, the Twin Rivers Hatchery has played a leading role in the reestablishment of burbot in the Kootenai River, ID, and British Columbia. We examined morphometric data (weight, length and condition factor), whole animal resting metabolic rate and the enzyme activity of lactate dehydrogenase, citrate synthase and 3-hydroxyacyl-CoA dehydrogenase to describe the baseline metabolic performance of cannibal and non-cannibal burbot. Taken together, our results demonstrated significant differences in the metabolic strategies of cannibal vs. non-cannibal burbot, where cannibals relied more heavily on carbohydrate metabolism and non-cannibals relied more heavily on glycolytic and lipid metabolism. This study demonstrates the need to reevaluate the traditional practice of removing cannibal fish in conservation hatcheries, as it may not be the ideal strategy of raising the most robust individuals for release. When natural habitat conditions cannot be restored due to permanent habitat alteration, prioritizing release of higher performing individuals could help achieve conservation goals.

## Teaser

Cannibalism is a common behaviour in fishes and is known to increase in aquaculture settings. The traditional aquaculture practice of reducing cannibalism to maximize yield may not be optimal for conservation-focused hatcheries, as there may be performance advantages of a cannibal feeding strategy.

## Introduction

Burbot, *Lota lota maculosa*, are the only freshwater member of the Gadidae family and exhibit a wide-reaching circumpolar distribution (McPhail and Lindsey, 1970). While burbot are not at risk as a species overall, select populations have experienced serious declines or extirpation (Stapanian *et al*., 2010). Burbot are rarely included in assessments and management plans (Paragamian, 2000), and efforts to curb local population declines often do not occur until after the population has already collapsed (Paragamian *et al*., 2008). The lower Kootenay population, located on the southern edge of British Columbia, is red-listed (S1) by the British Columbia Conservation Data Centre (B.C. Conservation Data Centre 2019). This population extends into the Kootenai River in Idaho, USA, and is the only endemic population of burbot in Idaho (Paragamian *et al*., 2000). The Kootenay/i^1^ population collapse is mainly attributed to construction of Libby Dam, near Jennings, MT, which altered water flow and elevated winter water temperatures above the spawning temperature range (Partridge, 1983; Paragamian *et al*., 2000). Burbot are also directly entrained in the dam turbines (Skaar *et al*., 1996). In addition to the dam, dikes and channelization along the river reduced shallow off-channel habitats and contributed to phytoplankton productivity declines (Daley *et al*., 1981). It is thought that the elimination of off-channel habitats contributed to the population decline of burbot, as the early life stages depend on plankton as a food source (Paragamian *et al*., 2011; Hardy and Paragamian, 2013). By 2003, declines in catch per unit effort indicated functional extirpation of burbot in the Kootenai River (KVRI Burbot Committee 2005).

In response to the local extirpation of burbot, in 2005 the Kootenai Valley Resource Initiative started a Burbot Conservation Strategy to develop methods to restore burbot in the region (KTOI 2017). The strategy has focused on using a conservation hatchery approach, located at the Twin Rivers Hatchery, to restore population levels in the region. In aquaculture settings, mitigating cannibalism is a primary issue. While cannibalism is a common phenomenon in wild fish populations, rates of cannibalism often increase in aquaculture due to the lack of available shelter, the inability to escape predation and high stocking densities (Baras and Jobling, 2002). Burbot exhibit high rates of intracohort cannibalism, which have been recorded as high as 45% (Trabelsi *et al*., 2011). Similar to other fish species, size gradation (i.e. grouping individuals of similar size) reduces rates of cannibalism in burbot (Barron *et al*., 2013), but it is a labour-intensive practice (Summerfelt *et al*., 2009; Kelly and Heikes, 2013; Lekang, 2013). Consistent with aquaculture standards, the Twin Rivers Hatchery has historically removed cannibals through size gradation to increase yield.

Cannibal burbot, however, are larger than non-cannibals, suggesting a potential physiological performance advantage of being a cannibal. Performance advantages associated with larger size in fishes include higher survival, growth and reproduction and are particularly important at younger life stages (Ware, 1975; Werner *et al*., 1983; Hutchings, 1991). Larger size could provide a post-release survival advantage through a reduction in predation risk (Krause *et al*., 1998). Furthermore, in rivers where damming and diking have resulted in low plankton abundance (e.g. Kootenai River; Daley *et al*., 1981), transitioning off of a planktivorous diet sooner may be advantageous for growth and survival. Cannibals may therefore have an advantage over their planktivorous siblings in a low-nutrient environment. Furthermore, it is thought that in oligotrophic environments cannibalism promotes the stability of the population as a whole (Nishimura and Hoshino, 1999; Nishimura and Isoda, 2004), suggesting that including cannibals in release programs would promote the Kootenai reestablishment goals.

Theoretically, the most effective method of reestablishing a wild population would be to release individuals with the highest fitness in the wild. Directly measuring the fitness of long-lived animals in the wild, however, is difficult, and we therefore must turn to other measures to act as proxies for fitness (e.g. physiological performance traits) (Pettersen *et al*., 2018). It is thought that for every performance trait, there is an underlying energetic cost spent by the animal (Sokolova *et al*., 2012). Therefore, under finite energy resources, individuals that can more efficiently budget energy may gain a performance advantage (Guderley and Pörtner, 2010). By examining energy use strategies, we can gain insight into potential performance differences between phenotypes.

In this study, we targeted metabolic strategies underlying performance to understand the energy use associated with burbot that utilize a cannibal feeding strategy compared to burbot that utilize a non-cannibal feeding strategy. We measured morphometrics (weight, length and Fulton’s condition factor) to gain insight into the energy allocation of each feeding strategy towards growth. To understand the relative aerobic, or oxidative, metabolic capacities of each feeding strategy, we measured whole-animal resting metabolic rate (RMR) and citrate synthase (CS) and 3-hydroxyacyl-CoA dehydrogenase (HOAD) enzyme activities. To understand the relative anaerobic, or glycolytic, metabolic capacities of each feeding strategy, we measured lactate dehydrogenase (LDH) enzyme activity. Taken together, the measures of oxidative and glycolytic capacities give insight into the overarching metabolic strategies of cannibal and non-cannibal burbot. We describe this as the baseline metabolic performance, as the burbot are under ideal hatchery conditions and not exposed to environmental stressors. We chose to examine baseline metabolic performance to identify any underlying metabolic differences based on feeding strategy alone. Given their larger size, we hypothesized that the metabolic strategy of cannibals was more efficient than that of their non-cannibal siblings, enabling the cannibals to out-perform the non-cannibals. Under the energy allocation hypothesis, a lower baseline energy requirement would allow more energy to be directed towards growth and reproduction (Gadgil and Bossert, 1970; Steyermark, 2002; Álvarez and Nicieza, 2005; Larivée *et al*., 2010). We therefore predicted that cannibals would show a lower mass-specific RMR and that cannibals would show a heightened reliance on aerobic metabolism, indicating the use of a more energetically efficient metabolic pathway.

## Materials and methods

### Experimental design

This study encompasses two experiments examining differences in the physiological performance of young cannibal burbot compared to non-cannibal burbot. Experiment 1, using whole fish samples collected on 7 August 2017, was our first study examining potential differences in cellular metabolic capacities between cannibal and non-cannibal burbot. Experiment 2, conducted from 23 to 27 July 2018, expanded on results found in Experiment 1 to further examine differences in metabolic enzymes between feeding strategies and to explore if there were metabolic differences on the organismal level.

### Fish rearing and collection

**Figure 1:**
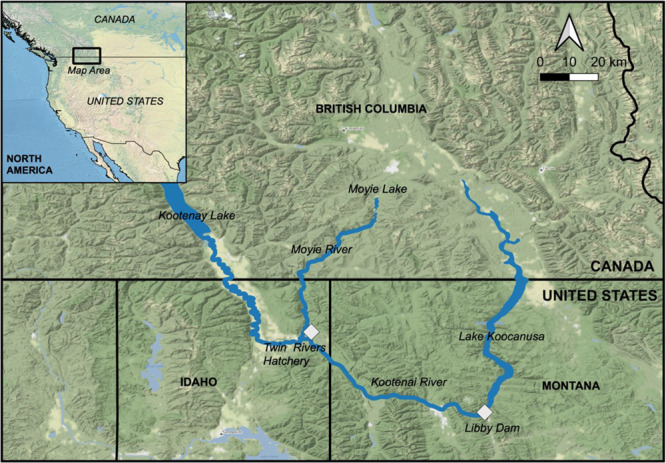
Primary areas of interest in our study, including adult broodstock sampling locations (the Kootenay River and Moyie Lake), the Twin Rivers Hatchery and the Libby Dam

All fish were spawned and raised at the Twin Rivers Hatchery in Bonners Ferry, ID, USA. Adult broodstock were caught from Moyie Lake, BC, Canada, and the Kootenai River, ID, USA, in January and spawned in February for both experimental years. Figure 1 depicts the primary locations of interest for this study, created using QGIS v3.8.1 (QGIS Development Team 2019), Natural Earth Data (Natural Earth 2019) and Open Street Map (Stamen Terrain 2012). Larval burbot were fed live rotifers *B. plicatilis* and *Artemia* spp. (San Francisco Bay and Great Salt Lake strains) a minimum of three times daily until the juvenile stage, when burbot were transitioned to a commercial cod diet and fed continuously at ~5% of body weight per day. Burbot were held in water temperatures that matched the incoming river water from the Kootenai River, slowly increasing from 3.5°C at hatch, to 10°C at the larval stage and to 14°C at the time of sampling, when the burbot had reached the juvenile stage. Fish were sampled in August of 2017 for Experiment 1 (at 1517 degree days, DD) and late July of 2018 for Experiment 2 (at ~1250 DD).

For both experiments, multiple families were sampled to incorporate genetic diversity in our study. Five families (named Ma, Mb, Mc, Md and Rv) were sampled for Experiment 1, while four families (named L1, L2, R1 and R2) were sampled for Experiment 2. Families Ma, Mb, Mc, Md, L1 and L2 are offspring of parents caught in Moyie Lake, while Rv, R1 and R2 are offspring of parents caught in the Kootenai River. All fish have a common genetic lineage from Moyie Lake. We used families originating in Moyie Lake and the Kootenai River to understand if parental habitat plays a role in modulating the physiological performance of different feeding strategies.

Cannibals were removed in the Mb, Mc and Rv tanks two to four times in 2017, following standard grading practices at the Twin Rivers Hatchery. Cannibals were removed on one occasion from the Ma and Md tanks in 2017 for sampling for a complementary study, but no other cannibals were removed for grading purposes. Cannibals were not removed from any of the 2018 tanks so that the Twin Rivers Hatchery could test the consequences of allowing cannibalism to run in a cohort. All families were held in one tank per family, and tank densities at the juvenile life stage were approximately 0.6 and 2.5 individuals per litre in 2017 and 2018 tanks, respectively. Hatchery technicians primarily identified cannibals as those with a tail of another burbot protruding from the mouth. Cannibals were also identified as those with black faeces protruding from the anus (hatchery feed is orange in colour, so non-cannibal burbot have orange faeces and cannibal burbot have black faeces).

For enzyme analyses, cannibal and non-cannibal burbot were sampled between 2:30 and 4:00 p.m. on 24 July 2018 and 25 July 2018, euthanized with a lethal dose of buffered tricaine methanesulfonate (500 mg/L, Syndel, Ferndale, WA, USA), flash frozen on liquid nitrogen and stored on dry ice during shipping until arrival at the University of California, Davis, where they were stored at −80°C until sample processing. For RMR sampling, tanks were removed from feed at 4:00 p.m. 2 days before sampling and individuals were isolated in holding tanks at 3:00 p.m. on the day before sampling, ensuring at least 36 h of fasting prior to oxygen consumption measurements. All fish handling procedures complied with the University of California, Davis Institutional Animal Care and Use Committee (protocol no. 19810).

### Morphometrics

For Experiment 1, morphometric data were limited to frozen wet weight of individuals. For Experiment 2, morphometric data consists of the fresh weight, length and Fulton’s condition factor. When fresh weights were collected, fish were dabbed dry and immediately weighed and the total length measured. Morphometric data reported for Experiment 2 were pooled to include fish used for respirometry experiments (*n* = 96) and those used for metabolic enzyme assays (*n* = 96), for a total of *n* = 192. Fulton’s condition factor was calculated as

$$ \mathrm{Condition}\ \mathrm{factor}=\frac{\mathrm{Weight}\ \left(\mathrm{g}\right)}{\mathrm{Total}\ \mathrm{length}\ {\left(\mathrm{cm}\right)}^3}\times 100 $$

### Cellular metabolic enzyme activity

In Experiment 1, 40 individuals (*n =* 4/feeding strategy/family, for a total of *n* = 20 per feeding strategy) were analyzed for LDH and CS activity. In Experiment 2, 96 individuals (*n* = 12/feeding strategy/family, for a total of *n* = 48 per feeding strategy) were analyzed for LDH, CS and HOAD activity. Protein homogenate was prepared as follows: individual fish were ground to a fine powder on liquid nitrogen and thoroughly mixed. A 50-mg sample of whole fish powder was homogenized in 500 μL potassium phosphate buffer (50 mM, pH 7.5) using a handheld homogenizer (Bio-Gen PRO200, PRO Scientific Inc., Oxford, CT, USA) at full speed for 15 s in Experiment 1 or a Bullet Blender (BBY24M, Next Advance Inc., Troy, NY, USA) at Speed 4 for 1 min in Experiment 2. All homogenates were held at ~4°C during homogenization. Homogenate was centrifuged at 4°C for two, 10-min rounds at 8000 rcf. Supernatant was collected and separated into aliquots for each enzyme assay and stored at −20°C until enzyme assays were conducted. All enzyme assays were run in triplicate in 96-well plates in a spectrophotometer (Synergy HT, BioTek Instruments Inc., Winooski, VT, USA) at 14°C. Activity was measured every minute for 30 min, and the maximum enzyme activity was calculated using a five-minute sliding window. Blank activity was measured for the first 15 min and calculated using a 5-min sliding window, before the addition of the start substrate, and subtracted from the maximum activity. All enzyme assays were optimized for burbot whole fish homogenate.

LDH activity, a measure of cellular glycolytic metabolic capacity, was determined by measuring the change in absorbance of NADH at 340 nm as it is converted to NAD^+^ in the presence of pyruvate. Assay buffer (52.5 mM Imidazole/HCl, pH = 7.5 at RT and 0.15 mM NADH) was added to 0.3 μg protein and read for background activity. Sodium pyruvate (2.64 mM in assay buffer) was then added to the reaction wells and immediately measured for maximum activity. CS activity, a measure of cellular oxidative metabolic capacity, was determined by measuring the change in absorbance of DTNB at 412 nm. Assay buffer (50 mM Imidazole/HCl, pH = 8.2 at RT and 0.4 mM Acetyl CoA) was added to 3 μg protein (Experiment 1) or 5 μg protein (Experiment 2) and read for background activity. Oxaloacetate (0.5 mM in assay buffer) was then added to the reaction wells and immediately measured for maximum activity. HOAD activity, a measure of lipid oxidation capacity, was determined by measuring the change in absorbance of NADH at 340 nm. Assay buffer (50 mM Imidazole/HCl, pH = 7.2 at RT and 0.3 mM NADH) was added to 20 μg protein and read for background activity. Acetoacetyl CoA (0.1 mM, aq.) was then added to the reaction wells and immediately measured for maximum activity. Reported concentrations for all assays are the in-well assay conditions. Every 96-well plate in all assays included positive and negative controls. All reagents were sourced from Sigma-Aldrich (Sigma-Aldrich, St. Louis, MO, USA). All reported enzyme activities are reported per μg protein, which was determined using the Pierce BCA Protein Assay Kit (Pierce Biotechnology, Rockford, IL, USA). Enzyme activity was calculated as:

$$ \mathrm{Activity}\ \left(\upmu \mathrm{mol}\ \mathrm{mg}\ {\mathrm{protein}}^{-1}{\min}^{-1}\right)=\frac{\mathrm{rA}}{\mathrm{L}\times \upvarepsilon \times \uprho}\times \frac{{\mathrm{V}}_{\mathrm{a}}}{{\mathrm{V}}_{\mathrm{s}}} $$

where rA is the rate of absorbance change (OD min^−1^), L is the optical path length (cm), ε is the extinction coefficient (6.22 OD mM^−1^ cm^−1^ for NADH and 14.15 OD mM^−1^ cm^−1^ for DTNB), *ρ* is the protein concentration of the homogenate (mg ml^−1^), *V*_a_ is the total volume of assay solution in each well (ml) and *V*_s_ is the volume of homogenate in each well (ml).

### RMR

Respirometry experiments measuring oxygen consumption were conducted to estimate the mass-specific RMR as part of Experiment 2. Three trials of eight fish were conducted every day for 4 days, for a total of 96 individuals (*n* = 12/feeding strategy/family for a total of *n* = 48 per feeding strategy). Fish were held in custom-built chambers with intermittent flow controlled using automated software (AutoResp v2.3.0 Loligo Systems, Viborg, Denmark). Oxygen consumption was measured using a fibre optic oxygen meter and probes (Witrox 4, Loligo Systems, Viborg, Denmark) and data acquisition software (DAQ-M, Loligo Systems, Viborg, Denmark). Each measurement cycle consisted of a flush, wait and measurement period as described in (Svendsen *et al*., 2016); each measurement period was ~10 min, and oxygen saturation did not fall below 80%. Fish were held in the respirometers for eight measurement cycles on average, or ~2 h and 40 min total. Because preliminary trials showed that two measurement periods were sufficient time for fish to recover from post-handling stress, the first two measurement periods were removed. RMR was calculated as the average of the three lowest slopes after the first two measures. The average RMR was then corrected for individual fish mass. The slopes of daily blanks were subtracted for the final reported RMR for each fish. Cannibal fish and non-cannibal fish were measured in 125-mL chambers and 22-mL chambers, respectively, to ensure that the fish weight to water volume ratios were between 1/20 and 1/100 as described in (Clark *et al*., 2013). Twin Rivers hatchery water was used in the respirometry chambers, and respirometry systems were fully submerged in tanks to maintain water temperature at rearing conditions (ca. 14°C). Fish were fasted for at least 36 h prior to measurement.

### Statistical analyses

All statistical analyses were performed in R using the R Studio interface (v3.5.2, R Core Team, 2018). Data were inspected for normality with Shapiro–Wilk tests and visually inspected for normality and homoscedasticity using Q–Q plots, distribution of residuals and residuals vs. fitted values using the ‘stats’ package (R Core Team, 2018), as advised in (Zuur *et al*., 2010). Datasets that were non-normal were transformed with appropriate Box–Cox transformations as identified with the ‘olsrr’ package (Hebbali, 2018) or analyzed using generalized linear models. Statistical relationships were analyzed using a two-way analysis of variance (ANOVA) using the base ‘stats’, the ‘lme4’ (Bates *et al*., 2015) and the ‘car’ (Fox and Weisberg, 2011) packages. Feeding strategy and family were tested as fixed main interactive effects and differences between groups were determined using Tukey’s HSD post hoc tests in the ‘lsmeans’ package (Lenth, 2016). Data visualization was conducted using the base ‘graphics’ package (R Core Team, 2018) and the ‘ggplot2’ package (Wickham, 2016). For the RMR experiment, one fish was removed from the dataset as it was flagged as having unusual oxygen consumption traces during the trial, had a low *r*^2^ for the RMR slopes and strongly skewed the model. Additionally, one of the experimental chambers showed a large effect on RMR values and was included as an indicator in the model. No values were removed from the morphometric or enzyme datasets. An *α* of 0.05 was used as the significance level for all statistical tests.

## Results

### Experiment #1

#### Morphometrics

**Figure 2:**
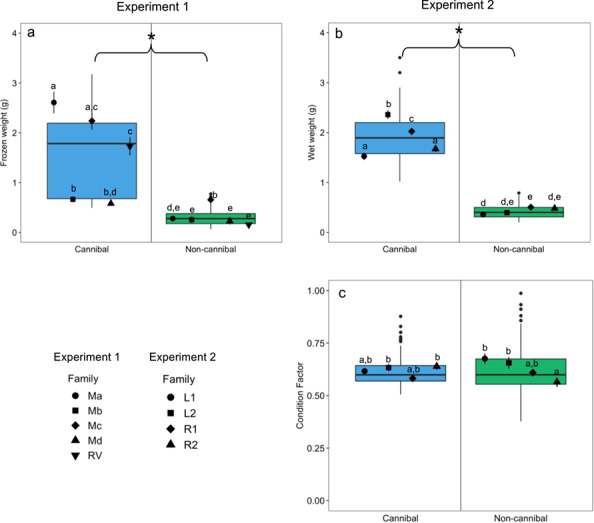
Morphometric data of juvenile cannibal (blue) and non-cannibal (green) burbot from Experiment 1 (**a**) in 2017 and Experiment 2 (**b**, **c**) in 2018 (*n* = 20/feeding strategy in Experiment 1 and *n* = 96/feeding strategy in Experiment 2). (a) Frozen weight (g) in Experiment 1, (b) fresh wet weight (g) in Experiment 2, (c) Fulton’s condition factor in Experiment 2. Data are presented as box plots with centreline, box and whiskers representing the median, inter-quartile range (IQR) and 1.5 times the IQR, respectively. Black points along the centreline indicate outliers between feeding strategies. Black symbols show the mean values (± s.e.m.) per family grouped within feeding strategy (*n* = 4/family/feeding strategy in Experiment 1 and *n* = 24/family/feeding strategy in Experiment 2). Letters indicate significant differences across all families and a star (*) indicates a significant difference between feeding strategies (*P* < 0.05)

Frozen fish weight was significantly affected by feeding strategy (*F*_1,30_ = 376.19, *P* < 0.01) and family (*F*_4,30_ = 39.80, *P* < 0.01), and the interaction between feeding strategy and family (*F*_4,30_ = 21.05, *P* < 0.01). Cannibals were significantly heavier than non-cannibals in every family (Fig. 2a).

#### Cellular metabolic enzyme activity

**Figure 3:**
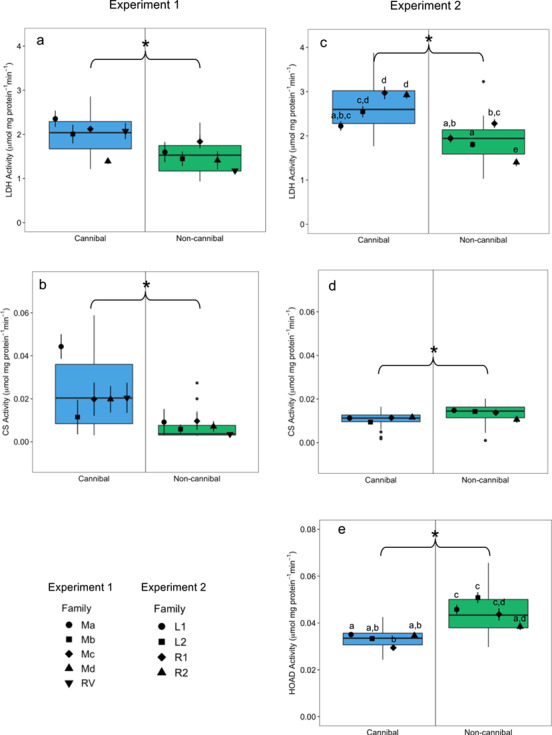
Cellular metabolic enzyme activities in whole body homogenate of juvenile cannibal (blue) and non-cannibal (green) burbot from Experiment 1 (**a**, **b**) in 2017 and Experiment 2 (**c**–**e**) in 2018 (*n* = 20/feeding strategy in Experiment 1 and *n* = 48/feeding strategy in Experiment 2). Panels (a, c) show lactate dehydrogenase (LDH) enzyme activity (μmol lactate mg protein^−1^ min^−1^), panels (b, d) show citrate synthase (CS) enzyme activity (μmol citrate mg protein^−1^ min^−1^), and panel (e) shows 3-hydroxyacyl-CoA dehydrogenase (HOAD) enzyme activity (μmol 3-hydroxyacyl-CoA mg protein^−1^ min^−1^). Data are presented as box plots with centreline, box and whiskers representing the median, inter-quartile range (IQR) and 1.5 times the IQR, respectively. Black points along the centreline indicate outliers between feeding strategies. The black symbols show the mean values (± s.e.m.) per family grouped within feeding strategy (*n* = 4/family/feeding strategy in Experiment 1 and *n* = 12/family/feeding strategy in Experiment 2). Letters indicate significant differences across all families and a star (*) indicates a significant difference between feeding strategies (*P* < 0.05)

LDH activity was significantly affected by feeding strategy (*F*_1,30_ = 23.28, *P* < 0.01) and family (*F*_4,30_ = 4.57, *P* < 0.01), but there was no significant interaction (*F*_4,30_ = 2.56, *P* = 0.058). Overall, LDH activity was significantly higher in cannibals than non-cannibals (Fig. 3a); however, when families were separated, LDH activity was only significantly higher in cannibals of the Rv family. CS activity was significantly affected by feeding strategy (*F*_1,38_ = 19.29, *P* < 0.01), but not family (*F*_4,34_ = 21.28, *P* = 0.195) and there was no interaction (*F*_4,30_ = 19.56, *P* = 0.624) (Fig. 3b). CS activity was significantly higher in cannibals than non-cannibals in Experiment 1.

**Figure 4:**
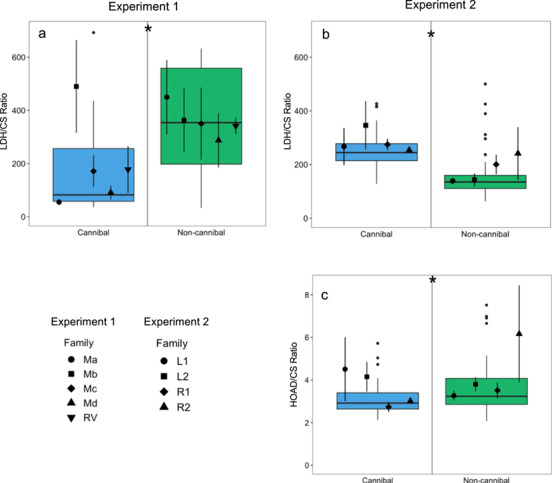
Cellular enzyme activity ratios in whole body homogenate of juvenile cannibal (blue) and non-cannibal (green) burbot from Experiment 1 (**a**) in 2017 and Experiment 2 (**b**, **c**) in 2018 (*n* = 20/feeding strategy in Experiment 1 and *n* = 48/feeding strategy in Experiment 2). Panels (**a**, **b**) show the LDH/CS ratios and panel (**c**) shows the HOAD/CS ratio. Ratios are of enzyme activity per milligram total protein. Data are presented as box plots with centreline, box and whiskers representing the median, inter-quartile range (IQR) and 1.5 times the IQR, respectively. Black points along the centreline indicate outliers between feeding strategies. The black symbols show the mean values (± s.e.m.) per family grouped within feeding strategy (*n* = 4/family/feeding strategy in Experiment 1 and *n* = 12/family/feeding strategy in Experiment 2). Letters indicate significant differences across all families and a star (*) indicates a significant difference between feeding strategies (*P* < 0.05)

The LDH/CS ratio was significantly affected by feeding strategy (*F*_1,30_ = 6.11, *P* = 0.019), but not family (*F*_4,30_ = 1.46, *P* = 0.236), and there was no interaction (*F*_4,30_ = 1.63, *P* = 0.191). Cannibal burbot had a lower LDH/CS ratio than non-cannibal burbot in Experiment 1 (Fig. 4a).

### Experiment 2

#### Morphometrics

Fish weight was significantly affected by feeding strategy (*F*_1,184_ = 2037.37, *P* < 0.01), family (*F*_3,184_ = 23.01, *P* < 0.01) and the interaction between feeding strategy and family (*F*_3,184_ = 18.90, *P* < 0.01). Cannibal fish were on average 125% larger than non-cannibal fish, although the magnitude of difference varied between families (Fig. 2b). Body condition, according to Fulton’s condition factor, was not significantly affected by feeding strategy (*F*_1,184_ = 0.0002, *P* = 0.9879), but was significantly associated with family (*F*_3,184_ = 4.05, *P* < 0.01) and the interaction of family and feeding strategy (*F*_3,184_ = 5.80, *P* < 0.01) (Fig. 2c). The R2 family was the only family to exhibit a significant difference in condition factor between feeding strategies, where cannibals had a significantly higher condition factor compared to non-cannibals. Non-cannibals exhibited a larger range in condition factor than cannibals.

#### Cellular metabolic enzyme activity

Feeding strategy had a significant effect on LDH activity (*F*_1,88_ = 129.80, *P* < 0.01); however, this relationship differed between families (*F*_3,88_ = 11.93, *P* < 0.01) as indicated by the significant interaction between feeding strategy and family (*F*_3,88_ = 14.76, *P* < 0.01). LDH activity was significantly higher in cannibals than non-cannibals in the L2, R1 and R2 families, but not in L1 (Fig. 3c). CS activity was also significantly affected by feeding strategy (*F*_1,88_ = 17.09, *P* < 0.01), but not by family (*F*_3,88_ = 1.73, *P* = 0.1664) or the interaction of feeding strategy and family (*F*_3,88_ = 2.39, *P* = 0.0737) (Fig. 3d). In contrast to Experiment 1, CS activity was significantly higher in non-cannibal burbot than cannibal burbot in Experiment 2. Feeding strategy had a significant effect on HOAD activity (*F*_1,94_ = 2.37, *P* < 0.01); however, this relationship differed between families (*F*_3,91_ = 2.01, *P* < 0.01) as indicated by the significant interaction between feeding strategy and family (*F*_3,88_ = 1.67, *P* < 0.01). HOAD activity was significantly higher in non-cannibals than cannibals in the L1, L2 and R1 families, but not in R2 (Fig 3e).

The LDH/CS ratio was significantly affected by feeding strategy (*F*_1,88_ = 45.28, *P* < 0.01), but not family (*F*_3,88_ = 1.43, *P* = 0.237) or the interaction of feeding strategy and family (*F*_3,88_ = 0.80, *P* = 0.493) (Fig. 4b). Cannibals had a significantly higher LDH/CS ratio on average than non-cannibals. Feeding strategy (*F*_1,88_ = 5.75, *P* < 0.02) and family (*F*_3,88_ = 3.85, *P* < 0.02) also had a significant effect on the HOAD/CS ratio, but there was no significant interaction between feeding strategy and family (*F*_3,88_ = 1.43, *P* = 0.238) (Fig. 4c). Non-cannibals had a significantly higher HOAD/CS ratio on average than cannibals.

#### RMR

**Figure 5:**
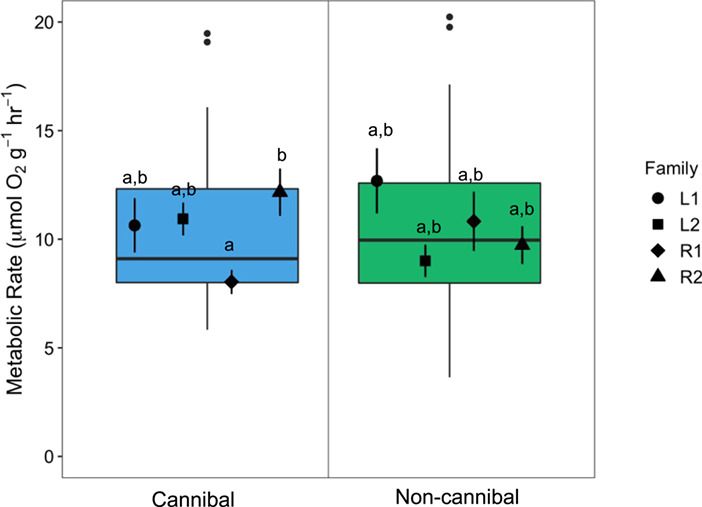
Mass-specific resting metabolic rate (μmol O_2_ g^−1^ h^−1^) of cannibal (blue) and non-cannibal (green) burbot from Experiment 2 in 2018. Data are presented as box plots with centreline, box and whiskers representing the median, inter-quartile range (IQR) and 1.5 times the IQR, respectively. Black points along the centreline indicate outliers between feeding strategies. The black symbols show the mean values (± s.e.m.) per family grouped within feeding strategy (*n* = 12/family/feeding strategy). Letters indicate significant differences across all families (*P* < 0.05). There was no significant difference in RMR between feeding strategies

There were no significant differences in mass-specific RMR based on feeding strategy (*X*^2^_1,85_ = 0.02, *P* = 0.882) or family (*X*^2^_3,85_ = 7.44, *P* = 0.059), but there was a significant interaction between feeding strategy and family (*X*^2^_3,85_ = 11.60, *P* < 0.01). RMR did not differ significantly between feeding strategies in any family (Fig. 5).

## Discussion

The aim of this study was to examine if cannibal and non-cannibal juvenile burbot differed in their baseline metabolic performance. Because the performance of animals is thought to be limited by the balance of energy supply and demand (Sokolova *et al*., 2012), we focused on examining differences in the metabolic capabilities between feeding strategies. By examining the oxidative and glycolytic capacities together, we characterized the baseline metabolic performance of cannibal and non-cannibal burbot.

### Morphometrics

The most overt difference observed between feeding strategies in this study was body size. Due to the physiological requirement of having a wide enough gape size to successfully cannibalize another fish, size heterogeneity is one of the most important factors associated with cannibalism in aquaculture (Smith and Reay, 1991; Hecht and Pienaar, 1993; Baras and Jobling, 2002). Heterogeneous growth has been shown to increase with grouping; when reared together, subordinate individuals grow slower than dominant individuals (Jobling and Wandsvik, 1983; Hofmann *et al*., 1999; Vera Cruz and Brown, 2007). In the present study, cannibals were removed throughout rearing in select families (i.e. Mb, Mc and Rv families in Experiment 1). Removing cannibals would be expected to reduce the social stress for the non-cannibals (e.g. through breaking the dominance hierarchy and reducing food competition) and allow them to reallocate energy previously spent on social stress towards growth (Volpato and Fernandes, 1994). Size heterogeneity, however, was maintained in tanks even when cannibals were removed, suggesting that social stress may not be an important driving factor behind the heterogeneous growth in cannibal and non-cannibal burbot.

A cannibal diet could provide a nutritional advantage compared to a non-cannibal diet, potentially explaining the increased growth in cannibal individuals. A previous study out of the Twin Rivers Hatchery found larval burbot lipid and protein content to be 10.10 and 70.12%, respectively (Jensen *et al*., 2011). In comparison to the commercial diet containing 14–15% lipid and 59–62% protein (GEMMA Micro, Skretting USA, UT, USA), a cannibal diet may be higher in protein and lower in lipid content. In the hatchery setting, we do not suspect that cannibal burbot are replacing their commercial diet, but that they are instead supplementing their diet with opportunistic cannibal events, presumably giving a supplement of extra protein. In the wild, larval burbot first feed on pelagic copepods and cladocerans, then transition to a primarily amphipod diet (Ryder and Pesendorfer, 1992). The diet of artemia and rotifers in the hatchery followed by solid commercial feed is quite different to the diet they would have in the wild, which may contribute to the high rates of cannibalism in the hatchery. There are many abiotic factors that affect cannibalism rates in aquaculture settings (e.g. light conditions, water temperature, shelter availability and density) that must be taken into consideration, in addition to food quality. We suspect that once released, the diet of cannibal burbot and non-cannibal burbot would overlap, but that cannibals would continue to cannibalize. The diet of burbot once released is unknown, however, and future studies examining dietary shifts between the feeding strategies once released and the proximate composition of each feeding strategy are needed to better understand the role that diet may play in shaping differential performance between cannibals and non-cannibals.

A study on wild adult burbot found cannibalism to be an important part of the adult burbot diet and, notably, that cannibal piscivores were significantly larger than non-cannibal piscivores (Gallagher and Dick, 2015). Given that the nutritional content of a similar fish species would not be expected to be significantly different than that of a conspecific, this suggests that the higher growth of cannibals cannot only be attributed to the nutritional content of eating fish flesh. Increased growth in cannibal fish compared to non-cannibal fish has been observed across species, including juvenile Atlantic cod, *Gadus morhua* (Folkvord and Otterå, 1993), Arctic char, *Salvelinus alpinus* (Amundsen, 1994), and larval walleye, *Stizostedion vitreum* (Li and Mathias, 1982), but the physiological mechanism driving the differential growth is not yet understood. Once on a cannibal growth trajectory, it is unlikely that a non-cannibal could ever ‘catch up’ in growth, as a positive feedback loop forms between growth rate and cannibalistic behaviour and further promotes heterogeneous growth (Baras and Jobling, 2002). Because of the canalization of becoming a cannibal (or not becoming a cannibal), identifying the mechanism underlying the increased growth in cannibals is an important area of future research. Examining potential sex-related differences in cannibal and non-cannibal burbot is also an area of future interest, as differential growth between sexes has been documented in other fishes (e.g. Imsland *et al*., 1997; Martin-Smith and Armstrong, 2002).

Observations from the Twin Rivers Hatchery suggest that when cannibal and non-cannibal burbot are held together, all individuals grow faster, not only the cannibals (Shawn Young, pers. obs.). Thus, it appears that there is an influence of cannibalism on the growth rate of not only the cannibal individuals but also the non-cannibal individuals, driving the growth dynamics of the entire cohort. One driving factor could be cannibals eating the underdeveloped individuals that are growing slower, which would likely die in a natural environment. By halting size gradation and allowing cannibalism to run in 2018, the Twin Rivers Hatchery was able to release juvenile burbot a month ahead of schedule due to the overall increased growth. The ability to release individuals earlier in the summer when river conditions are more favourable is paramount for a hatchery focused on the reestablishment of an extirpated species. The prospective benefits of halting size gradation and allowing cannibalism in rearing tanks to spur growth of cohorts deserves attention, as it could be used in conservation efforts of other populations of burbot or other species. Furthermore, halting size gradation would both reduce handing stress on the fish and the human labour involved in grading (Summerfelt *et al*., 2009; Kelly and Heikes, 2013; Lekang, 2013). Presumably, labour costs for the hatchery would also lower.

Given the pronounced weight differences between feedings strategies, we expected cannibals to exhibit a higher level of general health, as estimated using Fulton’s condition factor. Contrary to our expectations, there was only a significant difference in condition factor based on family and on the interaction of family and feeding strategy, suggesting that genetic variation between families is the primary driver of differences in condition factor. Because families were held in a single tank per family, tank effects cannot be excluded, but our results suggest that certain families may grow less optimally as compared to other families. While offspring from all families are released yearly by the Twin Rivers Hatchery, our results suggest examining condition differences between family groups may be important for any future long-term breeding programs.

### Cellular metabolic enzyme activity

Cannibal burbot demonstrated a heightened glycolytic anaerobic potential, as estimated from LDH enzyme activity, compared to non-cannibals in both experimental years. Heightened glycolytic anaerobic potential is thought to be associated with burst swimming activity in fishes (Somero and Childress, 1990). Cannibal burbot may be relying on burst swimming for hunting and attacking (Domenici and Blake, 1997). At the Twin Rivers Hatchery, we observed potential differential behavioural phenotypes between cannibal and non-cannibal burbot (pers. obs.), where the non-cannibals tended to continuously swim in the top portion of the water column and the cannibals hid beneath the water inflow until bursting out of hiding to attack non-cannibals. The behaviour of the cannibals is consistent with the ambush tactics documented in adult burbot in the wild (Hart, 1997; Pääkkönen, 2000). Examining the behavioural phenotypes of cannibal and non-cannibal burbot is an important area of further research, especially the potentially different burst swimming capacities between feeding strategies as they would presumably play an important role in successful foraging in the wild once released.

The higher capacity for fatty acid oxidation in non-cannibal burbot, as indicated by HOAD enzyme activity, indicates that non-cannibals could have relied more on fat stores to support ATP production. Cannibals, on the other hand, may have obtained sufficient fat content through their piscivorous diet and did not have to rely on metabolizing their own lipid stores. Increases in fatty acid oxidation can be associated with starvation, endurance training and/or environmental stress (Baldwin *et al*., 1972; McClelland, 2004; Morales *et al*., 2004). While the non-cannibals in the present study were certainly not starved, it is possible that they experienced food competition from the cannibals and ingested less food (Volpato and Fernandes, 1994). Our observation that non-cannibals tended to swim more constantly than cannibals, however, leads us to speculate that the heightened HOAD activity in non-cannibals reflects higher levels of endurance swimming as opposed to starvation. Non-cannibal burbot also demonstrated a higher HOAD/CS ratio compared to cannibals, indicating that a higher proportion of the non-cannibal aerobic metabolic pathway is dedicated towards lipid metabolism (Hochachka *et al*., 1983).

Notably, the oxidative capacity, as estimated from CS activity, showed an opposite pattern between experimental years. In Experiment 2, the non-cannibals demonstrated an 85% increase in oxidative capacity as compared to Experiment 1, which caused the relationship of oxidative capacity and feeding strategy to switch between experimental years. Our results could suggest higher swimming activities in non-cannibals in Experiment 2 compared to non-cannibals in Experiment 1, as oxidative capacity is known to increase with endurance training (Baldwin *et al*., 1972; McClelland *et al.*, 2004). Given that in Experiment 2 there was a constant presence of predators in the tanks due to holding cannibals and non-cannibals together, it is possible that the non-cannibals were increasing their swimming activities to avoid being eaten. Examining how the stocking conditions (and therefore the predator/prey dynamics of the tanks) influence the metabolism of young burbot is an important area of further research to better understand their metabolic strategies in relation to their environment. The different tank densities between years may have also contributed to the differences in CS activity across years. Alternatively, our results could suggest a higher growth rate in non-cannibals in Experiment 2, as oxidative capacity has also been shown to increase with increasing growth rates (Houlihan *et al*., 1993; Pelletier *et al*., 1993). In addition to the heightened growth rates observed in Experiment 2, the increased CS activity provides further evidence that stocking cannibals and non-cannibals together may increase the growth rate of entire cohorts. The opposite pattern in oxidative capacity drove the LDH/CS ratio to also switch between experimental years.

Given that all fish were larger in Experiment 2 than in Experiment 1, it is possible that we sampled the young burbot in different metabolic phases associated with ontogenetic and/or dietary shifts, as seen in other studies (Oikawa *et al*., 1991; Post and Lee, 1996; Finstad *et al*., 2006; Yagi *et al*., 2010). In yellow perch (*Perca flavescens*) and lake trout (*Salvelinus namaycush*), LDH activity levels demonstrated sharp drops at diet shifts (from planktivory to benthivory and from benthivory to piscivory) during growth (Sherwood *et al*., 2002). The drops in LDH activity are thought to be due to individuals requiring less time for foraging and attacking prey when the reward (i.e. the size of the prey) is larger, ultimately reducing burst swimming and associated LDH activity. In the present study, the non-cannibal individuals demonstrated the largest differences in LDH/CS between years; in Experiment 2, the non-cannibals may have been entering a different growth phase characterized by a higher reliance on aerobic metabolism. Given that the driving variable behind the opposite LDH/CS ratio was an increase in CS activity in non-cannibal burbot in Experiment 2, we interpret that the LDH/CS ratio in Experiment 2 may be due to a shift in metabolic strategy towards more aerobic metabolism in the non-cannibals.

Taken together, the elevated LDH activity in cannibals and elevated HOAD activity in non-cannibals suggests that cannibal burbot relied more heavily on carbohydrate metabolism and that non-cannibal burbot relied more heavily on lipid metabolism. The increase in CS activity in non-cannibal burbot between experimental years is evidence for a shift towards a more aerobic metabolic strategy, perhaps associated with dietary shifts through ontogeny, increased growth or differential stocking conditions.

### RMR

Despite their significantly larger size, cannibals did not exhibit a lower mass-specific RMR as predicted. We expected cannibals to exhibit a lower RMR based on the energy allocation hypothesis, that a lower baseline energy requirement allows more energy to be used for growth and reproduction (Gadgil and Bossert, 1970; Steyermark, 2002; Álvarez and Nicieza, 2005; Larivée *et al*., 2010). The significant interaction of feeding strategy and family on RMR indicates that there is some effect of feeding strategy on RMR once separated into families. In the R2 and L2 families, RMR was higher in cannibals than in non-cannibals and in the L1 and R1 families, RMR was higher in the non-cannibals than in the cannibals. This demonstrates that the environmental origin of the parental broodstock (Moyie Lake for the L1 and L2 families and the Kootenai River for the R1 and R2 families) was not a factor leading to differences in RMR. The average RMR of 10.4 μmol O_2_ g^−1^ h^−1^ (including cannibal and non-cannibal individuals) falls within a reasonable range of previously reported values for juvenile burbot (Fischer, 2000; Pääkkönen *et al*., 2003; Binner *et al*., 2008). Our results that a cannibal feeding strategy does not impart baseline aerobic metabolic differences on the whole-animal level do not follow patterns seen in previous studies on cannibalism and aggression in other fish species (Cutts *et al*., 1999; Lahti *et al*., 2002; Finstad *et al*., 2006). RMR does not capture the entirety of the metabolic phenotype of an animal and we hypothesize that differences could exist between feeding strategies in the maximum metabolic rate (MMR) and aerobic scope.

### Final remarks

The present study is the first to examine differential baseline metabolic performance between cannibal and non-cannibal burbot. Our results suggest that cannibal burbot have a heightened reliance on carbohydrate metabolism, while non-cannibal burbot rely more on lipid metabolism. Our study examined young burbot in a hatchery environment with abundant food resources and no inter-species predators. Understanding baseline metabolic performance differences under ideal conditions is valuable for predicting which individuals will demonstrate higher performance in more stressful natural environments. The metabolic strategies of cannibal and non-cannibal burbot are unknown once they are in the wild, as is their relative success. Future research comparing the long-term success of cannibal vs. non-cannibal individuals is necessary to determine the success of cannibal and non-cannibal burbot in a more ecologically accurate setting and to determine which metabolic strategy might be more optimal. In one scenario, cannibal burbot could outperform the non-cannibal burbot in the wild, because they were able to get on a faster growth trajectory, ultimately giving them a growth-advantage. In another scenario, however, the non-cannibal burbot may outperform the cannibal burbot if the environment is unable to provide the higher energy resources necessary for the cannibal feeding strategy.

This study directly contributes to the Kootenai Tribe’s burbot conservation aquaculture program and has broader implications for the conservation and commercial aquaculture efforts of other fish reestablishment programs. While conservation hatcheries remain controversial (e.g. due to the concern of maintaining wild genetic diversity, the threat of spreading disease, and the potential to release under-performing individuals), they are an important tool for reestablishing threatened populations (Anders, 1998; Brannon *et al*., 2004). Often, the concerns surrounding conservation hatcheries lead to them being used as a last resort, after populations are already functionally extinct (Anders, 1998). In the case of burbot in the Kootenai River, the Twin Rivers Hatchery has demonstrated early success at reestablishing a population, even after functional extinction in the region, and provides an example for the importance of using hatcheries to achieve conservation goals. Their program, led by the Kootenai Tribe of Idaho Native Fish Conservation Aquaculture Program, has had early success and burbot populations in the Kootenai River have increased from approximately 50 individuals in 2003 to approximately 45 000 in 2018 (Ross *et al*., 2018; >Siitari, 2018). The preliminary success has allowed the burbot fishery to open for the 2019–2021 seasons for the first time since 1992 (IDFG, 2020).

Due to the building of the Libby Dam and the construction of dykes, it is impossible (without significant wetland restoration including the removal of the dam and dykes) to restore the aquatic environment to historical conditions. When aquatic conditions cannot be restored, focusing on releasing physiologically robust individuals may help achieve reestablishment goals. Releasing physiologically robust individuals can give the highest chances of survival in a suboptimal environment. This study demonstrates the need to reevaluate the traditional practice of removing cannibal fish in conservation hatcheries of other fish species, as it may not be the ideal strategy of raising the most robust individuals for release.

## Footnotes

1 Spelled ‘Kootenay’ for Canadian waters and ‘Kootenai’ for American waters
